# Effects of Nano-CeO_2_ with Different Nanocrystal Morphologies on Cytotoxicity in HepG2 Cells

**DOI:** 10.3390/ijerph120910806

**Published:** 2015-09-02

**Authors:** Lili Wang, Wenchao Ai, Yanwu Zhai, Haishan Li, Kebin Zhou, Huiming Chen

**Affiliations:** 1 Chinese Academy of Inspection and Quarantine, Beijing 100123, China; E-Mails: llwang115@163.com (L.W.); aiwenchao126@163.com (W.A.); lihs@aqsiqch.ac.cn (H.L.); 2 Graduate University of Chinese Academy of Science, Beijing 100049, China; E-Mail: yyf2009319@foxmail.com

**Keywords:** nano-CeO_2_, cytotoxicity, apoptosis, MMP, ROS, GSH, antioxidant

## Abstract

Cerium oxide nanoparticles (nano-CeO_2_) have been reported to cause damage and apoptosis in human primary hepatocytes. Here, we compared the toxicity of three types of nano-CeO_2_ with different nanocrystal morphologies (cube-, octahedron-, and rod-like crystals) in human hepatocellular carcinoma cells (HepG2). The cells were treated with the nano-CeO_2_ at various concentrations (6.25, 12.5, 25, 50, 100 μg/mL). The crystal structure, size and morphology of nano-CeO_2_ were investigated by X-ray diffractometry and transmission electron microscopy. The specific surface area was detected using the Brunauer, Emmet and Teller method. The cellular morphological and internal structure were observed by microscopy; apoptotic alterations were measured using flow cytometry; nuclear DNA, mitochondrial membrane potential (MMP), reactive oxygen species (ROS) and glutathione (GSH) in HepG2 cells were measured using high content screening technology. The scavenging ability of hydroxyl free radicals and the redox properties of the nano-CeO_2_ were measured by square-wave voltammetry and temperature-programmed-reduction methods. All three types of nano-CeO_2_ entered the HepG2 cells, localized in the lysosome and cytoplasm, altered cellular shape, and caused cytotoxicity. The nano-CeO_2_ with smaller specific surface areas induced more apoptosis, caused an increase in MMP, ROS and GSH, and lowered the cell’s ability to scavenge hydroxyl free radicals and antioxidants. In this work, our data demonstrated that compared with cube-like and octahedron-like nano-CeO_2_, the rod-like nano-CeO_2_ has lowest toxicity to HepG2 cells owing to its larger specific surface areas.

## 1. Introduction

Cerium oxide nanoparticles (nano-CeO_2_) have a wide range of applications because of their desirable physical and chemical properties. They have been used extensively in catalysis, polishing glass, solid oxide fuel cells and many other applications [1,2]. It is precisely because of this extensive use in manufacturing industries, that concerns about the potential toxic effects of nano-CeO_2_ in humans and its impact on the environment have increased. Recently, nano-CeO_2_ has attracted considerable attention in the biomedical field. It can enter the general circulation system through the respiratory mucosal barrier, the gastrointestinal mucosal barrier and the skin barrier, after which it has access to all tissues and organs via the blood [3,4]. Many reports have demonstrated that nano-CeO_2_ is cytotoxic to various tissues and cell lines [5,6,7,8,9]. However, other researchers have postulated that nano-CeO_2_ is an antioxidant because it eliminates reactive oxygen species [10,11,12,13]. These two conflicting views are apparent on the safety of nano-CeO_2_. Size, morphology and concentration of nanoparticles are known to have significant effects on their biological responses [14,15,16], so the different biological effects of nano-CeO_2_ in these researches may be related to the size, morphology and/or concentration of the nanoparticles. Here, we aimed to compare the biological effects and toxicity of nano-CeO_2_ with different nanocrystal morphologies (cube-, octahedron-, and rod-like crystals) with an aim to find the safer morphology. 

Human HepG2 cells are commonly used as *in vitro* models to elucidate the pathophysiology of hepatocytes, the mechanism of cytotoxicity and in drug detection [17]. In the present study, we employed the HepG2 cell line to analyze changes in the morphology, viability, apoptosis, nuclear DNA, mitochondrial membrane potential (MMP), reactive oxygen species (ROS) and glutathione (GSH) of these cells in response to treatment with the three different nanocrystal morphologies of nano-CeO_2_.

## 2. Materials and Methods

### 2.1. Synthesis and Characterization of Nano-CeO_2_

Nano-CeO_2_ was synthesized by hydrothermal synthesis as described previously [14,18], to give cube-, octahedron-, or rod-like crystals. The phase analysis of the ceria nanoparticles was obtained by X-ray diffractometry (XRD, MSAL-XD2, Bragg Tech. Co., Ltd, Beijing, China) using Cu Kα radiation. The size and morphologies of nano-CeO_2_ were characterized by transmission electron microscopy (TEM, Philips CM30, Eindhoven, The Netherlands). The specific surface area of the nano-CeO_2_ was measured by Brunauer-Emmett-Teller (BET) area measurement [19].

### 2.2. Cell Culture

Human hepatocellular carcinoma HepG2 cells (catalog number pSC1046, purchased from the Beijing Institute of Genomics, Beijing, China) were maintained in Dulbecco’s modified Eagle’s medium (DMEM) containing 10% (v/v) fetal bovine serum (Hyclone, Logan, UT, USA), 100 μg/mL streptomycin (Sigma-Aldrich St. Louis, MO, USA) and 1000 IU/mL penicillin (Sigma-Aldrich). In all experiments, cells were incubated in a humid atmosphere at 37 °C with 5% (v/v) CO_2_.

### 2.3. Cellular Morphological and Internal Structure Observation

Exponentially growing HepG2 cells (1.0 × 10^5^ per well) were seeded into 6-well culture plates and incubated with various concentrations of nano-CeO_2_ (6.25, 12.5, 25, 50, 100 μg/mL) for 24 h. Changes in gross cellular morphology after each treatment were observed using a phase-contrast inverted biological microscope (IX71/IX2, Olympus, Tokyo, Japan). After that, the cells from each sample were harvested and washed with phosphate-buffered saline (PBS). After resuspension, the HepG2 cells were fixed in 3% malondialdehyde fluid. The samples were prepared for transmission electron microscopy (TEM) and the internal structure changes of cells were observed by TEM (JEOL JEM-1200EX, JEOL Ltd., Tokyo, Japan).

### 2.4. Apoptosis Assay

The HepG2 cells were treated with 6.25, 12.5, 25, 50, 100 μg/mL nano-CeO_2_ for 24 h. A total of 1.0 × 10^6^ cells from each treatment was collected using trypsin (without ethylenediaminetetraacetic acid (EDTA)), centrifuged (1000×*g* for 5 min), and washed twice with ice-cold PBS. Cells were then stained with fluorescein isothiocyanate (FITC)-labeled annexin V and propidium iodide (PI) according to the manufacturer’s instructions (Invitrogen, Carlsbad, CA, USA) and subjected to flow cytometric analysis (Beckman Coulter, Fullerton, CA, USA).

### 2.5. Measurement of Cell Count, and MMP, ROS and GSHLevels

A multiparametric cytotoxicity assay was performed using a cellomics high content screening (HCS) reagent HitKit as per the manufacturer’s instructions (Thermo Fisher Scientific Inc., Waltham, MA, USA). This kit measures cell count, MMP, ROS and GSH.

The HepG2 cells were placed on 96 well plates in the DMEM. On the second day, the cells were treated overnight with the 6.25, 12.5, 25, 50, 100 μg/mL nano-CeO_2_ and vehicle (0.1 % DMSO), respectively. After 24 h of incubation (37 °C, 5%CO_2_, 100% humidity), the media were removed and the cells were stained by fluorescent probes in the same culturing medium. The fluorescent probes were: Hoechst 33342 for cell count, tetramethylrhodamine methyl ester (TMRE) for mitochondrial membrane potential, 2′,7′-dichlorofluorescein (DCFH-DA) for ROS, and finally monochlorobimane (mBCL) for glutathione. Automated live-cell multispectral image acquisition was performed on a High Content Analysis (HCA) Reader (ArrayScan XTI, Thermo Fisher Scientific Inc.). The fluorescence images were captured according to the excitation and emission wavelengths of each probe: (1) 350 and 461 nm for Hoechst 33342 on channel 1; (2) 584 and 606 nm for TMRE on channel 4; (3) 504 and 529 nm for DCFH-DA on channel 3; (4) 380 and 461 nm for mBCL on channel 2. Image analysis was performed using HCS Studio™ 2.0 software (Thermo Fisher Scientific Inc.)

### 2.6. Antioxidant Activity Detection

Square-wave voltammetry (SWV) was performed to examine the ability of nano-CeO_2_ to eliminate the hydroxyl radical (·OH) as described by Xue using a CHI 440 electrochemical workstation (CH Instruments, Inc., Austin, TX, USA) [20]. The CO-Temperature Programmed Reduction (CO-TPR) (Micromeritics Autochem II 2920, Micromeritics, Norcross, GA, USA) was carried out to measure the oxidation-reduction properties of the nano-CeO_2_ using 10% CO/N_2_ at a flow rate of 25 mL/min and the furnace temperature up to 900 °C at a linear ramp of 15 °C/min. The samples were pretreated at 300 °C for 1 h before analysis.

### 2.7. Statistical Analyses

All data are presented as the mean ± the standard deviation (SD). Analysis of variance was used to compare values among all groups. Statistical analysis was undertaken with SPSS 10.0 (SPSS, Inc., Chicago, IL, USA) and *p* < 0.05 was considered as statistically significant (n = 3).

## 3. Results and Discussion

### 3.1. Characterization of the Nano-CeO_2_

**Figure 1:**
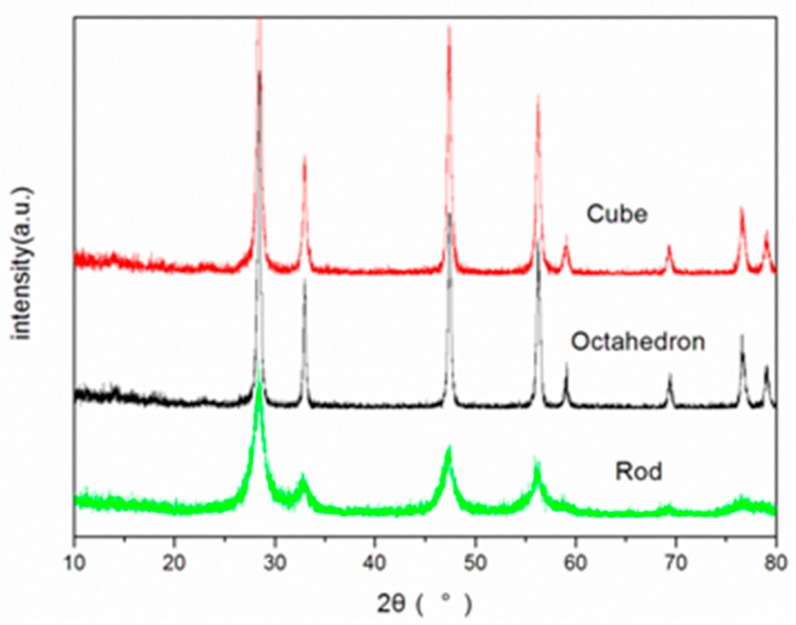
The XRD patterns of nano-CeO_2_ synthesized by hydrothermal processing.

**Figure 2:**
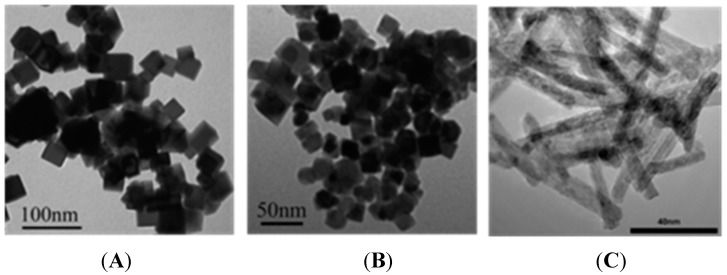
The TEM images of nano-CeO_2_ synthesized by hydrothermal processing. (**A**) Cubes; (**B**) octahedrons; (**C**) rods.

The nanoparticles were synthesized hydrothermally, and their XRD patterns, shown in Figure 1, indicate that these particles were composed of cerium oxide in the typical fluorite cubic structure (JCPDS 34-0394). The size and morphology of all nano-CeO_2_ are shown in Figure 2, and the results are in accordance with Table 1. Based on the BET results (Figure 2 and Table 2), the particle specific surface area decreases as the particle diameter increases.

**Table 1:** Three kinds of nano-CeO_2_ with different morphologies and sizes.

Number of nano-CeO_2_	Morphology	Size
1	Cube	20-50 nm in diameter
2	Octahedron	10-30 nm in diameter
3	Rod	8 nm in diameter and 100-400 nm in length

**Table 2:** Surface area of synthesized nano-CeO_2_.

Number of nano-CeO_2_	Morphology	The Specific Surface Area
1	Cube	18.9 m^2^·g^−1^
2	Octahedron	29.9 m^2^·g^−1^
3	Rod	83.2 m^2^·g^−1^

### 3.2. Morphological and Internal Structure Changes of Cultured HepG2 Cells Induced by Nano-CeO_2_

**Figure 3:**
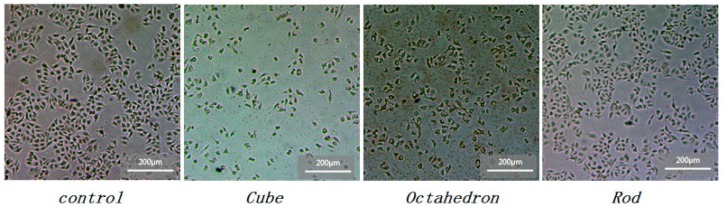
Photomicrographs of HepG2 cells with 50 μg/mL nano-CeO_2_ demonstrating significant changes in cellular morphology.

HepG2 cells were cultured in a full medium with one of the three types of nano-CeO_2_ at 6.25, 12.5, 25, 50, 100 μg/mL for 24 h. At the end of the culture, the cells with 100 μg/mL each type of nano-CeO_2_ were already damaged, while those cells cultured with 6.25, 12.5, and 25 μg/mL nano-CeO_2_ showed no significant changes in morphology when compared with the control group. For the 50 μg/mL treat group, there were marked differences in the morphology of the cells between each type of nanoparticle (Figure 3). The cell junctions were disrupted, cells shapes were altered, and a large number of dead cells were present in the culture supernatant.

**Figure 4:**
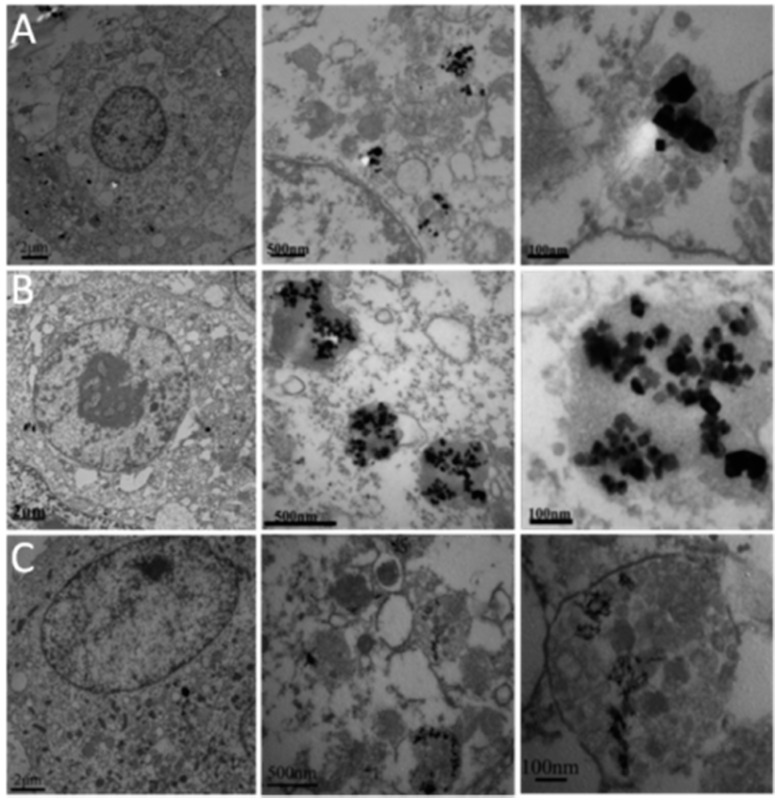
TEM images of HepG2 cells treated with 50 μg/mL nano-CeO_2_ for 24h. (**A**) Cubes; (**B**) octahedrons, (**C**) rods.

From these experiments, the dose (50 μg/mL) of treatment with nano-CeO_2_ was chosen for subsequent TEM examination (Figure 4). All three types of nano-CeO_2_ had aggregated and become localized in the lysosome and cytoplasm, accompanied by the disappearance of some lysosomal membranes. We believe the nano-CeO_2_ was internalized through the endocytosis pathway, but the exact mechanism is not clear and further research is needed.

### 3.3. Nano-CeO_2_ Induced Apoptosisin HepG2 Cells

**Figure 5:**
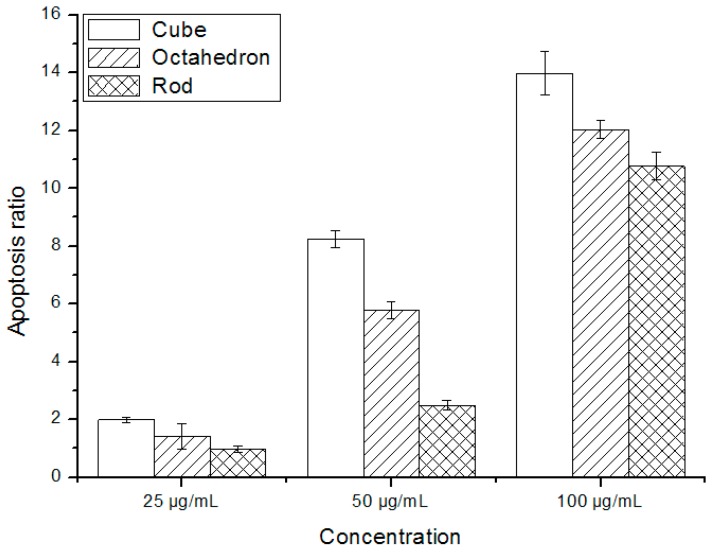
Percentage apoptosis of HepG2 cells after exposure to varying concentrations and types of nano-CeO_2_ for 24 h.

To investigate whether the observed cytotoxicity was due to apoptosis, the HepG2 cells were stained with annexin V conjugated to fluorescein isothiocyanate, which only binds to apoptotic cells. PI can penetrate into necrotic or late apoptotic cells, but does not label viable or early apoptotic cells. Meanwhile, annexin V, a protein with a high affinity for phosphatidylserine, binds to exposed phospholipids in apoptotic cells. In this study, those cells cultured with 6.25, and 12.5 μg/mL nano-CeO_2_ showed no significant changes in apoptosis compared with the control group. As shown in Figure 5, increased apoptotic rates were detected in 25, 50, and 100 μg/mL nano-CeO_2_-treated cells, and the increase was dose-dependent. The apoptosis percentage in the samples incubated without nanoparticles was 0.89%. The apoptotic proportion was higher for the cube-like nano-CeO_2_ than the other types. There were significantly greater rates of apoptosis in the nano-CeO_2_-treated groups than in the untreated controls (*p* < 0.01).

### 3.4. The Cell Count, and MMP, ROS and GSH Levels Induced by Nano-CeO_2_ in HepG2 Cells

High-content screening (HCS) is a novel method based on automated epifluorescence microscopy and acquired image analysis [21]. HCS has attracted attention as it can simultaneously measure multiple biomarkers in a single cell by multiplexed fluorescence measurements [22,23]. Application of HCS to organ-specific cell models provides deeper biological information suitable for better understanding the toxicity of compounds.

**Figure 6:**
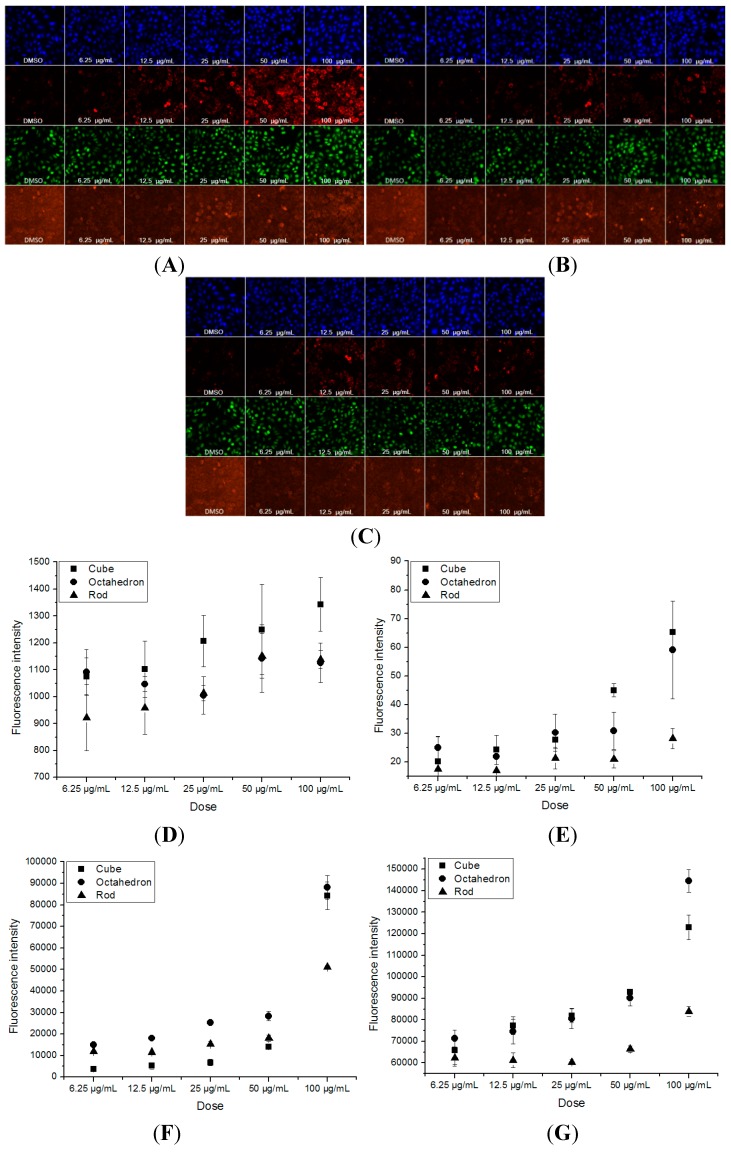
High-content screening of nano-CeO_2_ induced hepatotoxicity. HepG2 cells were treated with cube- (**A**), octahedron- (**B**), and rod-like (**C**) nano-CeO_2_ at various concentrations. The figures from top to bottom correspond to cellular images of cells count, MMP, ROS and GSH. Their normalized cellular fluorescence intensities were plotted as a function of treatment dose in cells count (**D**), MMP (**E**), ROS (**F**) and GSH (**G**).

Figure 6A–C shows concurrent observations of cell count, and MMP, intracellular ROS and GSH levels that were observed in the HepG2 cells after each treatment. The HepG2 cells were monitored as cellular aggregates because real-time monitoring is required for live HepG2 cells. The average cellular hepatotoxic response of the HepG2 aggregates was obtained from the selection of cellular regions and the average value of the fluorescence cellular images in Figure 6A–C. The fluorescence intensity in the y-axis of Figure 6D–G corresponds to the average fluorescence intensity of the observed HepG2 cells acquired through the cellular region selection of three different cell culture plates. The cellular fluorescence intensities obtained from three different cell culture plates were averaged and this was plotted as a function of the dose.

After the treatment of HepG2 cells with the three different types of nano-CeO_2_ at various concentrations, the fluorescence images of the HepG2 cellular aggregates are shown in Figure 6A–C. Then, a selection of identical cellular regions was executed with respect to all the fluorescence images of HepG2 aggregates acquired as a function of dose and their averaged fluorescence intensities were obtained. This process was applied to three different cell culture plates and the averaged value of the cellular fluorescence intensities obtained from individual cell culture plates was plotted as a function of dose as shown in Figure 6D–G. The fluorescence intensities in the y-axes of Figure 6D–G were normalized.

As shown in Figure 6A–C, the fluorescence intensities for cell count, MMP, ROS, and GSH present different amounts of changes, which vary with increasing concentration of nano-CeO_2_, for each morphology. These changes have certain dose-effect relationships, and are most sensitive in the cube-like groups. Hoechst 33342 allows the identification of individual cell nuclei, which permits a cell count by the nuclear area and allows subsequent analysis of the complementary staining to be conducted. A decrease in cell number indicates cell death and/or decreased cell proliferation. Nuclear shrinkage is typically a consequence of chromatin condensation and a sign of apoptotic cell death [24,25]. Additionally, the fluorescence intensity of Hoechst 33342 reflects the degree of apoptosis, because the apoptotic cells intake this dye more easily than living cells. Compared with the control (DMSO), the fluorescence intensity is distributed evenly and has no significant change in each group, except that the 100 μg/mL cube-like crystals induced a slight increase (Figure 6D). These results indicate that the cells did not undergo a lot of apoptosis or necrosis after each treatment. 

Mitochondria are essential for the function and survival of cells. They are responsible for the generation of ATP, Ca^2+^ uptake and storage, and the generation and detoxification of reactive oxygen species [24]. Mitochondrial membrane potential changes has previously been identified as a sensitive predictor of human drug-induced liver injury [26,27]. TMRE was used to measure mitochondrial function, as it accumulates in mitochondria that have maintained their inner membrane potential. As shown in Figure 6E, the fluorescence intensity of MMP increased in the HepG2 cells treated with the cube-like nano-CeO_2_ (the 100 μg/mL group was 2.8 times control). The rise in the MMP fluorescence intensity was gradual in the HepG2 cells treated with octahedron-like nano-CeO_2_ (the 100 μg/mL group was only 1.4 times the control). However, the rod-like nano-CeO_2_ showed a nearly constant MMP fluorescence intensity. The increase in TMRE brightness indicates mitochondrial hyperpolarization or a mitochondrial structural change that results in increased mitochondrial retention and/or fluorescence of TMRE [25]. Therefore, the most serious damage to the mitochondria was induced by the cube-like nano-CeO_2_ and the most minimal by the rod-like nano-CeO_2_. Due to the nanoparticles with various sizes and chemical compositions may attack mitochondria, which are redox-active organelles [12]. Nanoparticles may alter the production of ROS and interfere with antioxidant defenses, which will tend to induce oxidative stress [28,29,30,31,32]. To determine if nano-CeO_2_ induces oxidative stress in human hepatoma HepG2 cells, we measured the ROS and antioxidase (GSH) production. Accumulation of fluorescent compound 2′,7′-dichlorofluorescein (DCF), generated by intracellular oxidation of DHCF-DA, was used as an indicator of ROS generation, primarily H_2_O_2_. The production of ROS and MDA were found to increase after each treatment (Figure 6F,G). The maximum value of ROS was induced by 100 μg/mL of the cube-like nano-CeO_2_, which was about 143% of the control. The ROS for the octahedron-like nano-CeO_2_ was 120% of the control at 100 μg/mL. This level of ROS induced by the rod-like nano-CeO_2_ was similar to the control at the same concentration. (Figure 6F). In the cube-like group, the GSH value increased with dose from 106% to 207% of the control. At the highest concentration (Cmax), the octahedron-like group has a GSH value of 151% of control at maximum concentration, while the rod-like group reached only 130% at Cmax (Figure 6G).

Based on these results, the cube-like nano-CeO_2_ had an obvious effect on all four parameters. The octahedron-like had an effect on cell count, GSH and MMP, while the rod-like only increased GSH slightly. In summary, the toxicity of cube-like nano-CeO_2_ was the highest, the octahedron-like was lower, and the rod-like was the lowest.

### 3.5. Antioxidant Activity of Nano-CeO_2_

SWV has become one of the most sensitive methods for detecting DNA damage and antioxidant activity [20]. If nano-CeO_2_ can protect DNA from damage by eliminating hydroxyl radicals (·OH), the exposure of further guanine bases in DNA will be inhibited and the peak current of ruthenium (II) tris (2,2′-bipyridyl) ([Ru(bpy)3]^2+^) will not be increased [20].

**Figure 7:**
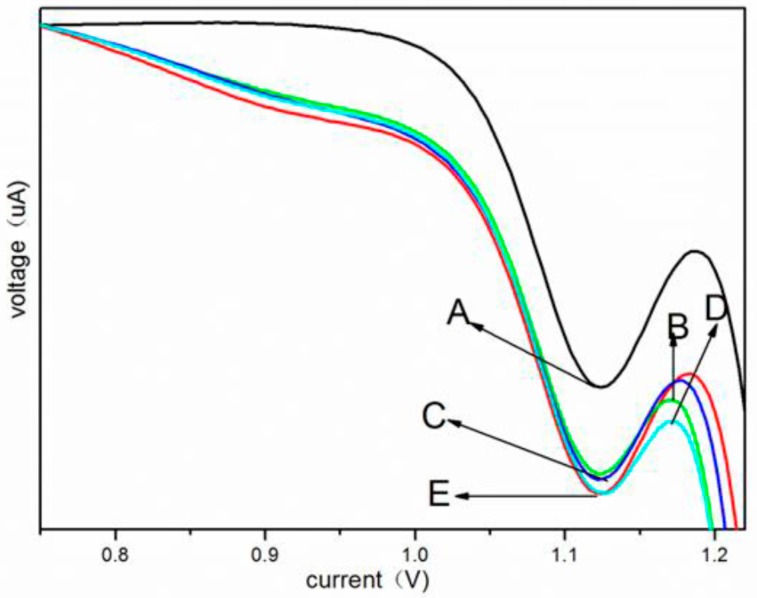
SWV scans in pH 7.0 PBS containing [Ru(bpy)_3_]^2+^ (50 uM) for PDDA/DNA films incubated for 20min: (**A**) without other agents, (**B**) with rod-like nano-CeO_2_, (**C**) with octahedron-like nano-CeO_2_, (**D**) with cube-like nano-CeO_2_, (**E**) with H_2_O_2_.

Our results (Figure 7) show the catalytic current of [Ru(bpy)3]^2+^ for an oxidation peak at around +1.10 V that is due to the electrocatalytic oxidation of [Ru(bpy)3]^2+^ by guanine bases in DNA. There is a major band for the intact double-stranded-DNA (lane A). When DNA was incubated in the solution of H_2_O_2_/Tris-HCl (pH 7.4), a new band (line E) could be seen that is due to DNA of different lengths, indicating that the DNA strands have been cut by·OH. When each type of nano-CeO_2_ (50 nM) was added to the system, the catalytic SWV current became smaller. The bands B and C came between lanes A and E, indicating the rod-like and octahedron-like nano-CeO_2_, respectively, protected and inhibited DNA damage by scavenging (·OH). Compared with bands B and C, band D nearly agrees with band E. This means that cube-like nano-CeO_2_ did not eliminate ·OH.

**Figure 8:**
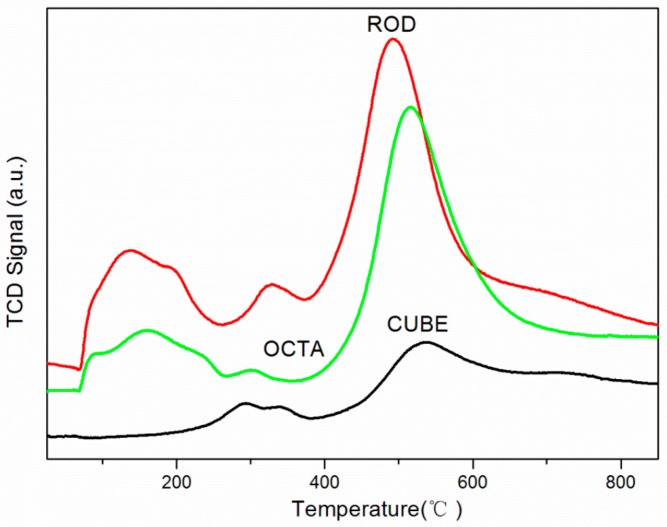
CO-TPR profiles of nano-CeO_2_.

Repeatable redox cycles between Ce^4+^ and Ce^3+^ occur on the surface of nano-CeO_2_ because of oxygen vacancies. The antioxidant activity of nano-CeO_2_ originates from its remarkable redox properties, which can be determined by the rate of the redox cycle [24]. The oxidation-reduction properties for the nano-CeO_2_ samples were determined by CO-TPR. The reduction peaks are shown in Figure 8. The low-temperature peak (<300 °C) was more important, for it is related to the reduction of surface oxygens. The results showed that the reduction peak of rod-like nano-CeO_2_ first appears at around 100 °C and the total area was the largest, indicating it had the largest amount of surface oxygen and was the easiest to reduce. In contrast, the first reduction peak of the cube-like nano-CeO_2_ was not detected until 300 °C with a very small peak. That is, the amount of surface oxygen was the least and it was the hardest to reduce. The octahedron-like was slightly easier to reduce than the rod-like but much harder than cube-like.

While the rate of nano-CeO_2_ oxidation was fast, the reduction of ceria was generally sluggish [33]. Thus, the redox properties and the antioxidant ability of nano-CeO_2_ were determined by its reducibility. In these results, the redox properties of the rod-like nano-CeO_2_ was the best, which meant that rod-like nano-CeO_2_ was the best at eliminating ROS. The cube-like was the worst with the octahedral somewhere in between. It is well known that the ability of nano-CeO_2_ to eliminate ROS is related to its redox chemistry [28]. Perhaps, the cytotoxicity of the rod-like was the lowest because it was the most able to eliminate ROS, and cube-like was the highest because it was least able. From these results, we speculate that the redox properties of nano-CeO_2_ may be an important factor in its cytotoxicity.

## 4. Conclusions

In summary, the *in vivo* cellular responses of HepG2 cells were measured following treatment with three types of nano-CeO_2_ with different morphologies. Different concentrations of nano-CeO_2_ had different toxicities on the HepG2 cells. The higher the concentration, the stronger the toxicity. At the same concentration, the nano-CeO_2_ that had a smaller surface area produced higher cytotoxicity and lower antioxidative property. Therefore, rod-like nano-CeO_2_ is a safer and more effective antioxidant when compared with octahedron and cube-like nano-CeO_2_.
